# Molecular screening of multidrug-resistance tuberculosis by a designated public health laboratory in Taiwan

**DOI:** 10.1007/s10096-017-3082-9

**Published:** 2017-08-24

**Authors:** H.-C. Lin, C.-L. Perng, Y.-W. Lai, F.-G. Lin, C.-J. Chiang, H.-A. Lin, R. Jou, T.-S. Chiueh

**Affiliations:** 1 Graduate Institute of Medical Sciences, National Defense Medical Center, Taipei, Taiwan Republic of China; 2 Division of Clinical Pathology, Department of Pathology, Tri-Service General Hospital, Taipei, Taiwan Republic of China; 3 School of Public Health, National Defense Medical Center, Taipei, Taiwan Republic of China; 4 Chest Hospital, Ministry of Health and Welfare, Tainan, Taiwan Republic of China; 5 Division of Infection, Department of Medicine, Tri-Service General Hospital SongShan Branch, Taipei, Taiwan Republic of China; 6 Tuberculosis Research Center, Taiwan Centers for Disease Control, No. 161, Kun-Yang Street, Taipei, 11561 Republic of China; 7 Institute of Microbiology and Immunology, National Yang-Ming University, Taipei, Taiwan Republic of China; 8 Department of Laboratory Medicine, Linkou Chang Gung Memorial Hospital, No. 5 Fu-Hsing Street, Kuei-Shan, TaoYuan City, 33305 Taiwan Republic of China

## Abstract

This manuscript describes our experience in early identifying MDR-TB cases in high-risk populations by setting up a single-referral molecular diagnosis laboratory in Taiwan. Taiwan Centers for Disease Control designated a single-referral laboratory to provide the GenoType MTBDR*plus* test for screening high-risk MDR-TB populations nationwide in 2012–2015. A total of 5,838 sputum specimens from 3,308 patients were tested within 3 days turnaround time. Compared with the conventional culture and drug susceptibility testing, the overall performance of the GenoType MTBDR*plus* test for detecting TB infection showed accuracy of 70.7%, sensitivity of 85.9%, specificity of 65.7%, positive predictive value of 45.5%, and negative predictive value of 93.3%. And the accuracy of detecting rifampin (RIF) resistance, isoniazid (INH) resistance, and MDR-TB (resistant to at least RIF and INH) were 96.5%, 95.2%, and 97.7%, respectively. MDR-TB contacts presented a higher rate of mutated codons 513–519, GenoType MTBDR*plus* banding pattern: *rpoB* WT3(−), and *rpoB* WT4(−) than the treatment failure group. The MDR-TB contact group also had a higher rate of *inhA* C15T mutation, banding pattern: *inh*A WT1(−), and *inhA* MUT1(+) than the recurrent group. Resistance profiles of MDR-TB isolates also varied geographically. The referral molecular diagnosis system contributed to rapid detection and initiation of appropriate therapy.

## Introduction

Tuberculosis (TB) is a major health challenge in Taiwan, where it is endemic [1]. The resurgence of TB in recent years has been shown to correlate with an increase in *M. tuberculosis* isolates which are resistant to one or more of the first-line treatment drugs [2]. A hospital-based study which evaluated 1961 Taiwanese TB patients between 2003 and 2007 showed that 11.7% of the patients were resistant to INH, 2.8% were resistant to RIF, 2.5% were resistant to EMB, and 11.1% were resistant to streptomycin (SM). The overall resistance to any drug was 19.1%, while 2% were multidrug-resistant (MDR; defined as resistant to at least INH and RIF) [3]. Other studies from Taiwan have shown that 10.1% of the *M. tuberculosis* strains were resistant to INH, 6.2% were resistant to RIF, 2.1% were resistant to EMB, and 9.8% were resistant to SM. In addition, 18.1% of these strains were resistant to any of the first-line drugs, and 4% were MDR [4–6]. The rates of MDR-TB among new cases and previously treated cases in Taiwan were 1.1% and 6–7% respectively [7].

The Taiwan Centers for Disease Control (TCDC) in accordance with WHO recommendations, implemented an MDR-TB management program (DOTS-plus program) in 2007 [4, 8]. However, the current standard care for MDR-TB has been shown to be associated with the emergence of extensively drug-resistant (XDR) TB [9, 10]. Earlier detection of MDR-TB cases and prompt initiation of appropriate treatment was therefore necessary for achieving the goal of DOTS-plus program.

Previous studies demonstrate drug resistance mutations of *rpoB, inhA*, and *katG* genes in *M. tuberculosis* [11–13]. Sequence analysis of the *rpoB* gene from 68 isolates demonstrated mutations in codons 531 (63.6%, TCG/TTG; 4.5%, TCG/TGG), 513 (9.1%, CAA/AAA), 533 (9.1%, CTG/CCG), 516 (4.5%, GAC/GTC), and 526 (4.5%, CAC/TGC) [14]. The most common *katG* mutations found in Taiwan were Arg463Leu (51%), Ser315Thr (29%), Ser315Asn (9.8%), and other loci (22%). However, the frequency of *inhA* gene mutations was low (2.4%) [15].

Recent advances in molecular techniques facilitate identifying *M. tuberculosis* and determining its drug susceptibility directly from clinical specimens [16–21]. The Xpert MTB/RIF system is an automated molecular test to detect *M. tuberculosis* and determine RIF resistance directly from sputum [22]. The GenoType MTBDR*plus* test recommended by WHO has been extensively used to detect *M. tuberculosis* complex and determine its resistance to RIF and INH [13, 18, 19, 23, 24]. The GenoType MTBDR*plus* test uses a combination of PCR amplification and reverse blotting with specific probes blotted on nitrocellulose strips to detect mutations of the *rpoB* (D516V, H526Y, H526D, S531 L), *katG* (S315 T), and *inhA* (C15T, A16G, T8C, and T8A) genes. Drug resistance is defined by the loss of any wild type probe’s signal or the presence of any mutant probe’s signal.

Although nucleotide mutations of the *rpoB*, *katG*, and *inhA* genes were previously shown to be associated with RIF and INH resistance respectively, there are currently no reports describing the correct interpretation of different banding patterns for this test. Additionally, since current published data on diagnostic performance of the GenoType MTBDR*plus* test are often limited in sample size, the diagnostic value of the test remains unclear in the general population or in specific groups of interest. In this study, we aimed to evaluate whether the GenoType MTBDR*plus* test is an adequate rapid test for diagnosis of pulmonary TB, and to determine drug resistance in TB high-risk populations.

## Methods

### Patient enrollment

We enrolled high-risk of MDR-TB individuals including default, treatment failure, relapse cases, inhabitants of aboriginal villages (Zhuoxi, Wanrong, and Xiulin villages in Hualien County) and suspects from the high TB burden counties (Lunbei in Yunlin County), MDR-TB contacts, and suspects staying in high MDR-TB burden countries for more than 1 month in the preceding year. A total of 5,838 sputum specimens were collected from 3,308 individual patients suspected with MDR-TB in Taiwan from March 2012 to December 2014. All specimens were subjected to acid-fast bacilli (AFB) staining, mycobacterial culture, and subsequent identification and drug susceptibility testing (DST) in authorized TB laboratories. Aliquots of the sputum sediments after NALC-NaOH (*N*-acetyl *L*-cysteine sodium hydroxide) decontamination were sent to the referral laboratory at Tri-Service General Hospital for molecular testing using the GenoType MTBDR*plus* test. All test results and patient data were uploaded to the TCDC website.

### Detection methods

#### Smear microscopy

Concentrated sputum smears were prepared by Petroff’s method. Both auramine O (AO) fluorescent dye and acid-fast stains were executed. Smear results were interpreted according to the guideline issued by the TCDC.

#### Isolation of *M. tuberculosis* and drug susceptibility testing (DST)

Both conventional solid agar and mycobacteria growth indicator tube (MGIT; Becton Dickinson, Sparks, MD, USA) media were used for isolation, and either conventional solid agar or MGIT was used for DST as previously described [25]. Detection of the MPB64 antigen and DST were performed as previously described [26, 27]. The sample was classified as drug resistant when the total number of colonies on the drug-containing medium were greater than 1% of the total number of colonies on the drug-free medium.

Growth of mycobacterial liquid cultures from sputum specimens was detected using an automated MGIT 960 apparatus (Becton Dickinson, Sparks, MD, USA) according to the manufacturer’s instructions. Positive cultures were evaluated by standard DST with INH and RIF using the MGIT 960 IR kit (Becton Dickinson) following the manufacturer’s instruction as previously described [28].

#### The GenoType MTBDR*plus* test for detecting drug resistance

Genomic bacterial DNA was extracted from 0.1 ml of the decontaminated sputum resuspension using the GenoLyse kit (Hain Lifescience, GmbH, Nehren, Germany) according to the manufacturer’s instructions. The PCR-amplified product DNA was used as a template in the GenoType MTBDR*plus* test (Hain Lifescience GmbH, Germany**)** which is based on reverse hybridization and an enzymatic color reaction. Drug resistance was interpreted according to the manufacturer’s instructions.

### Entry of results

Test results of non-MDR-TB were immediately reported on-line to the Infectious Disease Notification System of TCDC, since this national program project was conducted under commission to TCDC Ministry of Health and Welfare. Images of all GenoType MTBDR*plus* test strips were immediately scanned and emailed to TCDC for the second interpretation without annotation. Conventional mycobacterial culture and DST results were entered by the authorized TB laboratories to the Infectious Disease Notification System.

### Statistical analysis

Data for baseline patient characteristics were described as counts with percentages, except for age which was presented as mean and standard deviation. The multiple comparisons of percentages between pair-wise groups of various locations or reasons of screening were performed using the two-proportion-Z-test with Bonferroni correction. All hypothesis tests were two-sided with a significance level of 0.05. The results of the GenoType MTBDR*plus* test were summarized based on sensitivity, specificity, positive predictive value (PPV), negative predictive value (NPV), and accuracy. All statistical assessments were performed with the IBM SPSS software, version 22 (IBM Corp., Armonk, NY, USA).

## Results

### Characteristics of the screened subjects

More than one specimen from some patients were sent for examination in succession. MDR-TB cases were, however, only reported by individual patient instead of all specimens to avoid repetitive count on case number. Quality of specimens did result in variable results for some patients. Either discrepant negative result or repetitive positive result of the same patient was therefore omitted. No discrepant banding patterns of drug resistance were observed in specimens from the same individual. Finally, 3,308 cases were analyzed despite 5,838 specimens being tested.

Characteristics of screened individuals are summarized in Table 1. The average age of cases was 61.5 ± 17.3 years old. Most of specimens were collected from males (76.9%, 2,545/3,308). A majority of specimens were from relapse (46.9%, 1,552/3,308) and treatment failure (35.2%, 1,166/3,308) cases. Of the 3,308 screened cases, 23.0% were smear negative, and 9.9%, 34.7%, 15.5%, 7.7%, and 9.2% showed smear titers of scanty, 1+, 2+, 3+, and 4+, respectively. A total of 827 (25%) cases were *M. tuberculosis* culture positive.

**Table 1:** Characteristics of 3308 individuals suspected to have MDR-TB

		*N*= 3,308 cases
Subject characteristics		
Age (years)*		61.5 ± 17.3
Gender	Male	2,545 (76.9%)
	Female	763 (23.1%)
Reason for screening	Relapse	1,552 (46.9%)
	Treatment failure	1,166 (35.2%)
	Aboriginal inhabitants	379 (11.5%)
	Individual from high MDR-TB burden countries	76 (2.3%)
	Default (loss to follow-up)	69 (2.1%)
	MDR-TB contacts	66 (2.0%)
AFB Smear titer	Negative	762 (23.0%)
	Scanty	326 (9.9%)
	1+	1,147 (34.7%)
	2+	513 (15.5%)
	3+	255 (7.7%)
	4+	305 (9.2%)
*M. tuberculosis* isolation†		827 (25%)

Data are presented as counts and percentages except for *age which is presented as mean ± standard deviation

AFB, acid fast bacilli

† *M. tuberculosis* culture (+) among screened cases

### *M. tuberculosi*s isolation among the screened populations

*M. tuberculosis* isolation rates were 18.9% (242/1,278), 28.8% (302/1,047), and 28.8% (283/1,046) in 2012, 2013, and 2014, respectively (Table 2). The relapse group had a significantly higher isolation rate than the treatment failure group (29.4% vs 7.3%), but a lower rate than the aboriginal inhabitants groups, the group from high MDR-TB burden countries, the default group, and MDR-TB contacts (29.4% vs 41.7%, 78.9%, 43.5%, and 57.6%) (*p* < 0.05). The treatment failure group had the lowest isolation rate among all groups (*p* < 0.05).

**Table 2:** Isolation rates of *M. tuberculosis* among 3,308 screened populations by group

Year	Total	Relapse	Treatment failure	Aboriginal inhabitants	Individual from high MDR-TB burden countries	Default (Loss to follow-up)	MDR-TB contacts
Total	25% (827/3,308)	29.4% (456/1,552)	7.3% (85/1,166)	41.7% (158/379)	–	34.2% (13/38)	57.6% (38/66)
Year 2012 (March to Dec.)	18.9% (242/1,278)	20.8% (129/620)	8.1% (30/370)	25.7% (56/218)			43.8% (14/32)
Year 2013	28.8% (302/1,047)	33.1% (164/496)	9.3% (36/389)	57.3% (55/96)	83.9% (26/31)	44.4% (8/18)	76.5% (13/17)
Year 2014	28.8% (283/983)	37.4% (163/436)	4.7% (19/407)	72.3% (47/65)	75.6%(34/45)	69.2% (9/13)	64.7% (11/17)

Data are represented as percent (*n*/*N*) for given specific screening reason and time period. *n*, number of subjects with TB isolation (+); *N*, total subjects for given specific group and time period

### Comparison of GenoType MTBDR*plus* test results with conventional mycobacterial DST results

Using the conventional mycobacterial isolation results as gold standard, the GenoType MTBDR*plus* test detected TB with 85.9%, 65.7%, 45.5%, 93.3%, and 70.7% of sensitivity, specificity, PPV, NPV, and accuracy, respectively (Table 3).

**Table 3:** Performance of the GenoType MTBDR*plus* test (*n* = 3308)

GenoType MTBDR*plus*		*M. tuberculosis* isolation		Sen.	Spe.	PPV	NPV	Accuracy
		Yes	No					
TB	Yes	710	851	85.9%	65.7%	45.5%	93.3%	70.7%
	No	117	1,630					

Abbreviations: TB, tuberculosis; Sen., sensitivity; Spe., specificity; PPV, positive predictive value; NPV, negative predictive value; MDR, multidrug resistance

The sensitivity, specificity, PPV, NPV, and accuracy of the GenoType MTBDR*plus* test were 92.1%, 97.3%, 85.3%, 98.6%, and 96.5%, respectively for detecting RIF resistance; were 77.9%, 99.8%, 99.1%, 94.6%, and 95.2%, respectively for detecting INH resistance; and were 82.7%, 99.7%, 97.1%, 97.7%, and 97.7%, respectively for detecting MDR (Table 4).

**Table 4:** The performance of the GenoType MTBDR*plus* test in predicting mycobacterial drug resistances (*n* = 710*)

GenoType MTBDR*plus*		Corresponding DST results†		Sen.	Spe.	PPV	NPV	Accuracy
		R	S					
RIF resistance	R	93	16	92.1%	97.3%	85.3%	98.6%	96.5%
	S	8	567					
INH resistance	R	109	1	77.9%	99.8%	99.1%	94.6%	95.2%
	S	31	543					
MDR	R	67	2	82.7%	99.7%	97.1%	97.7%	97.7%
	S	14	601					

*710 patients with Genotype TB (+) and culture *M. tuberculosis* isolation (+)

†Only 684 patients with completed DST results

Abbreviations: R, resistance; S, susceptible; Sen., sensitivity; Spe., specificity; PPV, positive predicted value; NPV, negative predicted value; INH, isoniazid, RIF, rifampicin; MDR, multidrug resistance to at least INH and RIF

### Associations of *rpoB* mutations with reason for screening and geographic area

Analysis of the *M. tuberculosis* genotype from each group and location showed that patients in the MDR contact group had a significantly higher proportion of *rpoB* WT3(−) and *rpoB* WT4(−) cases (33.3% and 28.6% respectively) compared to the treatment failure group (both 0%). Patients in the aboriginal inhabitants group had a significantly higher proportion of *rpoB* WT8 (−/weak) (100%) compared to patients in the treatment failure group (45.5%). Additionally, there was a significantly lower proportion of *rpoB* MUT3 (+) mutations in the south (35.1%) compared to the north (68.8%), east (77.8%), and central areas (50%). There was a significantly higher proportion of *rpoB* WT8 (+) in the south compared to the north (Table 5).

**Table 5:** The association of *rpoB* mutation with reason for screening and geographic area (*n* = 101)

		Reason for screening (*n*, %)						Geographic area (*n*, %)				(*n*, %)
		Recurrent	Failed (treatment failure)	Aboriginal inhabitants	High MDR-TB burden countries	Default (lost to follow-up)	MDR-TB contacts	North	Central	South	East	Outlying islands
*rpoB* MUT1	+	1 (2.5)	0 (0)	0 (0)	0 (0)	0 (0)	0 (0)	0 (0)	0 (0)	1 (2.7)	0 (0)	0 (0)
	−	39 (97.5)	22 (100.0)	9 (100.0)	6 (100.0)	3 (100.0)	21 (100.0)	32 (100.0)	22 (100.0)	36 (97.3)	9 (100.0)	1 (100.0)
*rpoB* MUT2A	+	2 (5.0)	2 (9.1)	0 (0)	1 (16.7)	0 (0)	0 (0)	2 (6.3)	1 (4.5)	1 (2.7)	1 (11.1)	0 (0)
	−	38 (95.0)	20 (90.9)	9 (100.0)	5 (83.3)	3 (100.0)	21 (100.0)	30 (93.7)	21 (95.5)	36 (97.3)	8 (88.9)	1 (100.0)
*rpoB* MUT2B	+	3 (7.5)	2 (9.1)	0 (0)	0 (0)	1 (33.3)	1 (4.8)	1 (3.1)	1 (4.5)	5 (13.5)	0 (0)	0 (0)
	−	37 (92.5)	20 (90.9)	9 (100.0)	6 (100.0)	2 (66.7)	20 (95.2)	31 (96.9)	21 (95.5)	32 (86.5)	9 (100.0)	1 (100.0)
*rpoB* MUT3	+	22 (55.0)	8 (36.4)	7 (77.8)	4 (66.7)	1 (33.3)	11 (52.4)	22 (68.8)	11 (50.0)	13 (35.1)*	7 (77.8)	0 (0)
	−	18(45.0)	14 (63.6)	2 (22.2)	2 (33.3)	2 (66.7)	10 (47.6)	10 (31.2)	11 (50.0)	24 (64.9)	2 (22.2)	1 (100.0)
*rpoB* WT1	+	39 (97.5)	22 (100.0)	9 (100.0)	6 (100.0)	3 (100.0)	21 (100.0)	32 (100.0)	22 (100.0)	36 (97.3)	9 (100.0)	1 (100.0)
	−	1 (2.5)	0 (0)	0 (0)	0 (0)	0 (0)	0 (0)	0 (0)	0 (0)	1 (2.7)	0 (0)	0 (0)
*rpoB* WT2	+	37 (92.5)	19 (86.4)	9 (100.0)	5 (83.3)	3 (100.0)	20 (95.2)	31 (96.9)	22 (100.0)	31 (83.8)	8 (88.9)	1 (100.0)
	−	3 (7.5)	3 (13.6)	0 (0)	1 (16.7)	0 (0)	1 (4.8)	1 (3.1)	0 (0)	6 (16.2)	1 (11.1)	0 (0)
*rpoB* WT3	+	33 (82.5)	22 (100.0)	9 (100.0)	6 (100.0)	2 (66.7)	14 (66.7)	30 (93.7)	17 (77.3)	30 (81.1)	8 (88.9)	1 (100.0)
	−	7 (17.5)	0 (0)	0 (0)	0 (0)	1 (33.3)	7 (33.3)†	2 (6.3)	5 (22.7)	7 (18.9)	1 (11.1)	0 (0)
*rpoB* WT4	+	35 (87.5)	22 (100.0)	9 (100.0)	6 (100.0)	2 (66.7)	15 (71.4)	30 (93.7)	18 (81.8)	32 (86.5)	8 (88.9)	1 (100.0)
	−	5 (12.5)	0 (0)	0 (0)	0 (0)	1 (33.3)	6 (28.6)†	2 (6.3)	4 (18.2)	5 (13.5)	1 (11.1)	0 (0)
*rpoB* WT5	+	38 (95.0)	22 (100.0)	9 (100.0)	6 (100.0)	3 (100.0)	21 (100.0)	32 (100.0)	21 (95.5)	36 (97.3)	9 (100.0)	1 (100.0)
	−	2 (5.0)	0 (0)	0 (0)	0 (0)	0 (0)	0 (0)	0 (0)	1 (4.5)	1 (2.7)	0 (0)	0 (0)
*rpoB* WT6	+	38 (95.0)	22 (100.0)	9 (100.0)	6 (100.0)	3 (100.0)	21 (100.0)	32 (100.0)	21 (95.5)	36 (97.3)	9 (100.0)	1 (100.0)
	−	2 (5.0)	0 (0)	0 (0)	0 (0)	0 (0)	0 (0)	0 (0)	1 (4.5)	1 (2.7)	0 (0)	0 (0)
*rpoB* WT7	+	34 (85.0)	14 (63.6)	9 (100.0)	5 (83.3)	2 (66.7)	19 (90.5)	28 (87.5)	20 (90.9)	26 (70.3)	8 (88.9)	1 (100.0)
	−	6 (15.0)	8 (36.4)	0 (0)	1 (16.7)	1 (33.3)	2 (9.5)	4 (12.5)	2 (9.1)	11 (29.7)	1 (11.1)	0 (0)
*rpoB* WT8	+	14 (35.0)	12 (54.5)	0 (0)	2 (33.3)	1 (33.3)	9 (42.9)	7 (21.9)	8 (36.4)	21 (56.8)¶	2 (22.2)	0 (0)
	−	26 (65.0)	10 (45.5)	9 (100.0)†	4 (66.7)	2 (66.7)	12 (57.1)	25 (78.1)	14 (63.6)	16 (43.2)	7 (77.8)	1 (100.0)

^*^ *p* < 0.01, significant difference compared with the location in the north^*^

^*^ *p* < 0.01, significant difference compared with the reason for screen in failed^†^

### Associations of *inhA/katG* mutations with reason for screening and gengraphic area

Patients in the MDR contact group had a significantly higher proportion of *inhA* MUT1(+) cases (47.8%) compared to patients in the recurrent group (18.2%). However, the MDR contact group had a significantly lower proportion of *inhA* WT1(+) cases (47.8%) compared to patients in the recurrent group (83.6%). Interestingly, there were no significant differences in the prevalence of the *inh*A/*kat*G mutations in the different geographical areas (Table 6).

**Table 6:** The association of *inhA/katG* mutations with reason for screening and geographic area (*n* = 140)

			Reason for screening (*n*, %)					Gengraphic area (*n*, %)			
		Recurrent	Failed (treatment failure)	Aboriginal inhabitants	High MDR-TB burden countries	Default (lost to follow-up)	MDR-TB contacts	North	Central	South	East
*inhA* MUT1	+	10 (18.2)	9 (28.1)	7 (35)	3 (42.9)	0(0)	11 (47.8)*	11 (23.4)	11 (33.3)	13 (29.5)	5 (31.3)
	−	45 (81.8)	23 (71.9)	13 (65)	4 (57.1)	3 (100.0)	12 (52.2)	36 (76.6)	22 (66.7)	31 (70.5)	11 (68.7)
*inhA* MUT2	+	0 (0)	0 (0)	0 (0)	0 (0)	0 (0)	0 (0)	0 (0)	0 (0)	0 (0)	0 (0)
	−	55 (100.0)	32 (100.0)	20 (100.0)	7 (100.0)	3 (100.0)	23 (100.0)	47 (100.0)	33 (100.0)	44 (100.0)	16 (100.0)
*inhA* MUT3A	+	3 (5.5)	0 (0)	0 (0)	0 (0)	0 (0)	0 (0)	3 (6)	0 (0)	0 (0)	0 (0)
	−	52 (94.5)	32 (100.0)	20 (100.0)	7 (100.0)	3 (100.0)	23 (100.0)	44 (94)	33 (100.0)	44 (100.0)	16 (100.0)
*inhA* MUT3B	+	0 (0)	0 (0)	0 (0)	0 (0)	0 (0)	0 (0)	0 (0)	0 (0)	0 (0)	0 (0)
	−	55 (100.0)	32 (100.0)	20 (100.0)	7 (100.0)	3 (100.0)	23 (100.0)	47 (100.0)	33 (100.0)	44 (100.0)	16 (100.0)
*inhA* WT1	+	46 (83.6)	23 (71.9)	13 (65)	4 (57.1)	3 (100.0)	11 (47.8)*	35 (74.5)	22 (66.7)	32 (72.7)	11 (68.8)
	−	9 (16.4)	9 (28.1)	7 (35)	3 (42.9)	0 (0)	12 (52.2)	12 (25.5)	11 (33.3)	12 (27.3)	5 (31.2)
*inhA* WT2	+	51 (92.7)	32 (100.0)	20 (100.0)	7 (100.0)	3 (100.0)	23 (100.0)	43 (91.5)	33 (97.6)	44 (94.7)	16 (97.7)
	−	4 (7.3)	0 (0)	0 (0)	0 (0)	0 (0)	0 (0)	4 (8.5)	0 (0)	0 (0)	0 (0)
*katG* MUT1	+	24 (43.6)	20 (62.5)	11 (55)	4 (57.1)	3 (100.0)	12 (52.2)	27 (57.4)	13 (39.4)	23 (52.3)	11 (68.8)
	−	31 (56.4)	12 (37.5)	9 (45)	3 (42.9)	0 (0)	11 (47.8)	20 (42.6)	20 (60.6)	21 (47.7)	5 (31.2)
*katG* MUT2	+	0 (0)	0 (0)	0 (0)	0 (0)	0 (0)	0 (0)	0 (0)	0 (0)	0 (0)	0 (0)
	−	55 (100.0)	32 (100.0)	20 (100.0)	7 (100.0)	3 (100.0)	23 (100.0)	47 (100.0)	33 (100.0)	44 (100.0)	16 (100.0)
*katG* WT	+	34 (61.8)	14 (43.8)	8 (40)	3 (42.9)	0 (0)	11 (47.8)	22 (46.8)	20 (60.6)	24 (54.5)	4 (25)
	−	21 (38.2)	18 (56.2)	12 (60)	4 (57.1)	3 (100.0)	12 (52.2)	25 (53.2)	13 (39.4)	20 (45.5)	12 (75)

^*^ *p* < 0.01, significant difference compared with the reason for screen in recurrent

No significant difference was derived among geographic areas

## Discussion

Although clinical practice guidelines have recommended the routine use of nucleic-acid amplification testing for evaluating MDR-TB since 1996, clinicians and laboratory personnel have not implemented the recommendation widely because of the cost. Taiwan is a moderate-TB-burden country (annual TB incidence: 49.4/100,000 population in 2015), with approximately 120 new MDR-TB cases reported annually. It has been reported that 1.1% of the Taiwanese annual new TB cases has MDR-TB. Recent data have shown that MDR status was significantly associated with poor long-term outcome, and MDR-TB patients had significantly shorter survival times compared to non-MDR-TB patients [29]. Screening only high-risk patients with the GenoType MTBDR*plus* test would significantly reduce the cost burden, since patients without significant risk factors can continue to be screened using traditional methods. We therefore established a referral molecular diagnosis laboratory for nation-wide screening of MDR-TB using a cost-effective strategy. Our data were consistent with previous studies on the performance of the GenoType MTBDR*plus* tests [13, 18, 19, 23, 24, 29].

Although mycobacterial culture and conventional DST remain the gold standards for diagnosis of TB and determining drug susceptibility, they are so time-consuming and technically demanding that effective and timely treatment can be compromised. The GenoType MTBDR*plus* test enables rapid diagnosis of TB and detection of INH and or RIF resistance. Drug resistance data showed that the GenoType MTBDR*plus* test was highly consistent with conventional DST assays [30–33], and the results could be reported within 3 working days. Earlier clinical management of patients identified with MDR-TB could certainly reduce the risk of transmission. Additionally, recent studies demonstrated the use of the GenoType MTBDR*plus* test to confirm the presence of *M. tuberculosis* in HIV-infected patients, and in AFB-negative smears with positive TB-PCR results [34, 35]. Single drug-resistant mycobacteria are mostly not cultivatable because of inhibition by the residual anti-TB drugs in specimens, and administration of effective treatment can be compromised. Negligence of the mono-resistant mycobacteria could potentially result in selecting for MDR-TB due to insufficient efficacy of the combined regimen. Indeed, erroneous treatment strategies and previous TB therapy were recently shown to be highly correlated with acquisition of MDR-TB, and each additional treatment episode doubled the risk of MDR-TB [36].

Rapid diagnosis of MDR-TB using molecular methods was therefore introduced for blocking secondary transmission as early as possible. In this study, rapid molecular testing yielded positive results in 851 cases (25.7%) which were negative by a conventional culture method. This might be because patients were receiving therapy, or because of low bacilli load in the specimens. Additionally, a total of 11 MDR-TB cases were positive by molecular rapid testing but negative by conventional culture. This could be due to the low concentration drug resistance missed by conventional DST. Furthermore, 117 cases which were negative by molecular rapid testing but positive by conventional culture had scanty smear results. This might be due to the detection limit of the GenoType MTBDR*plus* test, and is an inherent disadvantage of this generation of the GenoType MTBDR*plus* test, which can only be used in specimens with positive sputum smears. Therefore, drug-resistant gene sequencing is recommended to confirm wild-type results which may be missed by the GenoType MTBDR*plus* test.

A major advantage of the GenoType MTBDR*plus* test is its ability to detect mutations associated with RIF and INH resistance. A previous study which detected the presence of *rpoB*, *inhA*, and *katG* mutations using the GenoType MTBDR*plus* test reported the benefits of using this assay as an initial screen to reduce the delay in initiating treatment, and to guide the recommendation for first- and second-line DST [37]. Early initiation of an appropriate treatment regimen is especially important, since this may be a critical factor to reduce the number of patients lost to follow-up [38]. However, to the best of our knowledge, ours is the first study to analyze the association between *rpoB*, *inhA*, and *katG* mutations and the reason for screening, as well as geographical area. Our data showed that patients in the MDR contact group had a higher proportion of *rpoB* WT3(−) and *rpoB* WT4(−) (denoting mutations in codons 513–519), and patients in the aboriginal inhabitants group had a higher proportion of *rpoB* WT8 (−) (denoting mutations in codons 530–533) compared to the treatment failure group. The MDR contact group also had a lower prevalence of *inh*A WT1(+) and a higher prevalence of *inhA* MUT1(+) (denoting a C15T mutation) compared to the recurrent group. Analysis of mutations associated with location showed that there was a significantly lower proportion of *rpoB* MUT3(+) and a significantly higher proportion of *rpoB* WT8(+) mutations in the south compared to the north.

## Conclusions

We demonstrated the diagnostic value of the GenoType MTBDR*plus* test for detection of MDR-TB. In addition to its high accuracy, the major advantage of the GenoType MTBDR*plus* test is the ability to reduce possible secondary transmission of MDR-TB by virtue of earlier diagnosis with satisfactory sensitivity. This system is still being implemented, and provides MDR-TB identification service nation-wide. We expect that it will help to reduce the annual incidence of MDR-TB in Taiwan.
